# The Influence of Pre-Procedural Imaging and Cystic Duct Cholangiography on Endoscopic Transpapillary Gallbladder Drainage in Acute Cholecystitis

**DOI:** 10.3390/diagnostics11071286

**Published:** 2021-07-16

**Authors:** Junya Sato, Kazunari Nakahara, Yosuke Michikawa, Ryo Morita, Keigo Suetani, Akihiro Sekine, Yosuke Igarashi, Shinjiro Kobayashi, Takehito Otsubo, Fumio Itoh

**Affiliations:** 1 Department of Gastroenterology and Hepatology, St. Marianna University School of Medicine, Kawasaki 216-8511, Kanagawa, Japan; j2sato@marianna-u.ac.jp (J.S.); y2michikawa@marianna-u.ac.jp (Y.M.); roadrunnner58@yahoo.co.jp (R.M.); k2suetani@marianna-u.ac.jp (K.S.); akihiro.sekine@marianna-u.ac.jp (A.S.); y2igarashi@marianna-u.ac.jp (Y.I.); fitoh@marianna-u.ac.jp (F.I.); 2 Department of Gastroenterological and General Surgery, St. Marianna University School of Medicine, Kawasaki 216-8511, Kanagawa, Japan; koharubiyori@marianna-u.ac.jp (S.K.); otsubo@marianna-u.ac.jp (T.O.)

**Keywords:** acute cholecystitis, endoscopic transpapillary gallbladder drainage, cystic duct, computed tomography, magnetic resonance imaging

## Abstract

Endoscopic transpapillary gallbladder drainage (ETGBD) for acute cholecystitis is challenging. We evaluated the influence of pre-procedural imaging and cystic duct cholangiography on ETGBD. Patients who underwent ETGBD for acute cholecystitis were retrospectively examined. The rate of gallbladder contrast on cholangiography, the accuracy of cystic duct direction and location by computed tomography (CT) and magnetic resonance cholangiopancreatography (MRCP), and the relationship between pre-procedural imaging and the technical success of ETGBD were investigated. A total of 145 patients were enrolled in this study. Gallbladder contrast on cholangiography was observed in 29 patients. The accuracy of cystic duct direction and location (proximal or distal, right or left, and cranial or caudal) by CT were, respectively, 79%, 60%, and 58% by CT and 68%, 55%, and 58% by MRCP. Patients showing gallbladder contrast on cholangiography underwent ETGBD with a significantly shorter procedure time and a lower rate of cystic duct injury. No other factors affecting procedure time, technical success, and cystic duct injury were identified. Pre-procedural evaluation of cystic duct direction and location by CT or MRCP was difficult in patients with acute cholecystitis. Patients who showed gallbladder contrast on cholangiography showed a shorter procedure time and a lower rate of cystic duct injury.

## 1. Introduction

Acute cholecystitis is an inflammation of the gallbladder that is most often caused by gallstones [1]. The risk factors of cholelithiasis have been reported as obesity, female, pregnancy, and others [2]. Although acalculous cholecystitis represents only 5–10% of all cases of cholecystitis in adults, acalculous cholecystitis is the most frequent form of acute cholecystitis in childhood [3]. Acute cholecystitis is usually diagnosed by ultrasound sonography or computed tomography (CT) in 3–10% of patients who experience abdominal pain [4,5,6]. The standard therapy for acute cholecystitis is laparoscopic cholecystectomy [7]. According to the Tokyo Guidelines 2018 [8], gallbladder drainage is considered in patients with moderate or severe acute cholecystitis when emergency cholecystectomy is not suitable.

Percutaneous transhepatic gallbladder drainage (PTGBD) and endoscopic transpapillary gallbladder drainage (ETGBD) are effective methods to drain the gallbladder. ETGBD is especially performed in patients who cannot undergo PTGBD for reasons such as ongoing antithrombotic therapy, ascites, or an anatomically inaccessible location [9,10]. However, ETGBD is technically challenging. Previous reports showed that the technical success rate of ETGBD was 64–100%, which is lower than that reported for PTGBD [11]. The most difficult part of ETGBD may be the process of inserting a guidewire into the gallbladder through the cystic duct. In addition, ETGBD may specifically result in cystic duct injury as an intraprocedural adverse event when a device, such as a guidewire, cannula, or stent, is advanced through the cystic duct.

To overcome and prevent these problems, pre-procedural imaging examinations such as CT or magnetic resonance cholangiopancreatography (MRCP) and cholangiography of the cystic duct might be important. However, only a few studies have investigated this topic. Therefore, we retrospectively evaluated the influence of pre-procedural imaging and cholangiography of the cystic duct in patients who underwent ETGBD for acute cholecystitis.

## 2. Materials and Methods

### 2.1. Patients

This study was conducted at the Department of Gastroenterology and Hepatology of the St. Marianna University School of Medicine. Patients with acute cholecystitis who underwent ETGBD between January 2011 and December 2019 were retrospectively reviewed. Patients were excluded if they met any of the following conditions: (1) no contrast-enhanced CT performed before ETGBD, (2) endoscopic transpapillary gallbladder aspiration performed without stent placement, or (3) cystic duct direction and location not assessable by endoscopic retrograde cholangiopancreatography (ERCP). All patients provided written informed consent for the procedure. This study was approved by the Institutional Review Board of St. Marianna University School of Medicine (approval number: 4382).

### 2.2. ETGBD Procedure

We used a duodenoscope (JF-260V, TJF-260V, or TJF-Q290V; Olympus Medical Systems, Tokyo, Japan) and performed bile duct cannulation using conventional contrast or wire-guided cannulation. After cannulation, cholangiography was performed to assess the shape of the common bile duct and determine whether the cystic duct showed contrast. A hydrophilic guidewire (e.g., 0.035-inch Radiforcus, Terumo Co. Ltd., Tokyo, Japan; 0.025/0.035-inch NaviPro, Boston Scientific, Natick, MA, USA) was passed through the cystic duct. After the guidewire was inserted into the gallbladder, the guidewire was changed to a stiff type. We then inserted a 7-French (Fr) tapered catheter with side holes (MultiFunction Catheter; Gadelius Medical, Tokyo, Japan) into the gallbladder over the guidewire to suction out the infected bile. Finally, we placed a plastic stent or naso-drainage catheter for ETGBD. The stents used for endoscopic gallbladder stenting (EGBS) were as follows: a new stent designed for EGBS (GBest-N stent; Hanaco Medical Co., Saitama, Japan) [12], double-pigtail stents (Advanix; Boston Scientific, Natick, Massachusetts, United States; SET-ERBD-72 stent; Hanaco Medical Co., Saitama, Japan; CX-T stents; Gadelius Medical, Tokyo, Japan; PBD-203 stent; Olympus Medical Systems, Tokyo, Japan; and Zimmon biliary stent; COOK Japan, Tokyo, Japan), and a straight-type stent (Through pass; Gadelius Medical, Tokyo, Japan). In cases where ETGBD could not be performed successfully, we usually performed endoscopic biliary stenting (EBS) or 6-Fr endoscopic nasobiliary drainage (ENBD) for bile duct drainage during the same ERCP session. All ETGBDs were performed under the supervision of an expert with experience in over 1000 ERCP procedures.

### 2.3. Measurements

**Figure 1:**
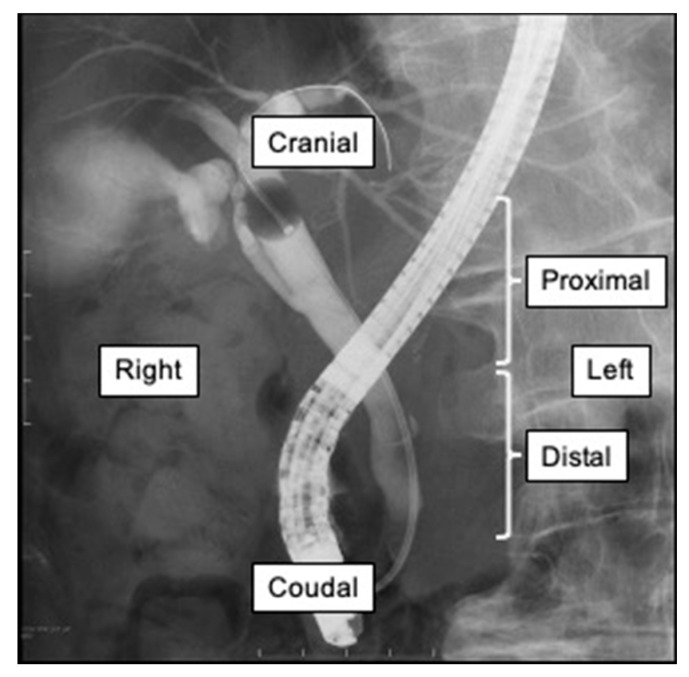
Cystic duct direction and location. The example shows the proximal, right, and cranial branches of the cystic duct.

We retrospectively examined the patients’ background, cystic duct contrast on cholangiography, cystic duct direction and location, ETGBD procedure time, technical success of ETGBD, cystic duct injury during ETGBD, technical success rate of ETGBD, and the accuracy of cystic duct direction and location by CT and MRCP. The diagnosis and severity of acute cholecystitis were assessed according to the Tokyo Guidelines 2018 [13]. The final cystic duct directions and locations were judged on the basis of ERCP findings (Figure 1). The CT, MRCP, and ERCP images were checked by three experts (J.S., K.N., and Y.M.) in pancreatobiliary endoscopy, and the directions and locations of the cystic duct in each modality were determined. Technical success of ETGBD was defined by the placement of the tip of the stent or drainage catheter in the gallbladder. Cystic duct injury was defined by dislocation of the guidewire or cannula from the cystic duct lumen, as confirmed by fluoroscopic imaging during ERCP. Furthermore, we assessed the relationship between pre-procedural images and procedural results of ETGBD, such as procedure time, technical success, and cystic duct injury.

### 2.4. Statistical Analysis

Categorical variables were compared using Fisher’s exact test. Continuous variables are presented as median (range) and compared using the Mann–Whitney U-test. Additionally, *p* values < 0.05 were regarded as denoting significance. Statistical analyses were performed using R version 3.4.1 software (R Foundation, Vienna, Austria).

## 3. Results

### 3.1. Patient Characteristics

**Figure 2:**
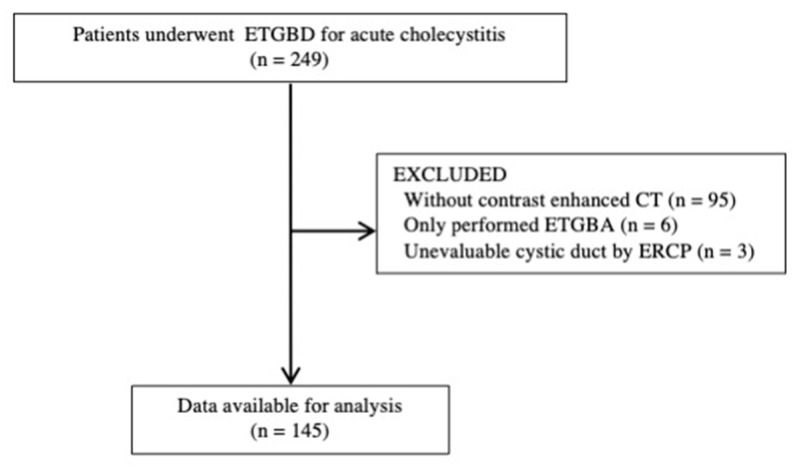
Patient flowchart. ETGBD: endoscopic transpapillary gallbladder drainage; ETGBA: endoscopic transpapillary gallbladder aspiration; CT: computed tomography, ERCP: endoscopic retrograde cholangiopancreatography.

Between January 2011 and December 2019, 249 consecutive patients underwent ETGBD for acute cholecystitis. Among them, 104 patients were excluded because they did not undergo contrast-enhanced CT (95 patients), only underwent endoscopic transpapillary gallbladder aspiration (6 patients), or had an unevaluable cystic duct on ERCP (3 patients). A total of 145 patients were enrolled in this study (Figure 2). The reasons for selecting ETGBD rather than cholecystectomy were as follows: receiving antithrombotic therapy in 56 patients, presence of common bile duct stones in 41 patients, severe comorbidities in 36 patients, and insufficient performance status in 11 patients. Gallstones were the most common cause of cholecystitis (82%) (Table 1). The causes of acalculous cholecystitis were malignant obstruction in eight patients, metal stent in six patients, plastic stent in one patient, embolism of gallbladder artery in one patient, liver cyst in one patient, sclerosing cholangitis in one patient, and unknown in eight patients. Stone impactions at the cystic duct and gall neck were observed in 17 and 21 patients, respectively. Mild, moderate, and severe cholecystitis was diagnosed in 69 patients (48%), 55 patients (38%), and 21 patients (14%), respectively.

### 3.2. Cholangiography and ERCP

During cholangiography from the distal bile duct, the cystic duct alone showed contrast in 31 patients (21%), and the cystic duct and gallbladder showed contrast in 29 patients (20%) (Table 2). The most common cystic duct directions and locations were the proximal, right, and cranial branches in 114, 101, and 141 patients, respectively. The mean procedure time for ETGBD was 50 min, and the technical success rate was 88%. Cystic duct injury was observed in 16 patients (11%). The dislocated devices were the guidewire in 11 patients and the cannula in 5 patients.

### 3.3. Accuracy by CT and MRCP

The accuracy of the cystic duct direction and location (proximal or distal, right or left, and cranial or caudal) was 79%, 60%, and 58%, respectively, by CT (Table 3). MRCP was performed in 31 patients. The accuracy of the cystic duct direction and location (proximal or distal, right or left, and cranial or caudal) was 68%, 55%, and 58%, respectively, by MRCP.

### 3.4. Relationship between Pre-Procedural Images and ETGBD

The predictors associated with the outcomes of ETGBD were investigated using univariate analyses (Table 4). Patients showing gallbladder contrast on cholangiography underwent ETGBD with a significantly shorter procedure time than those without gallbladder contrast on cholangiography. No other factors affecting the procedure time, technical success, and cystic duct injury rate of ETGBD were identified. Although the ETGBD procedure time and success rate showed no statistically significant differences, the procedure tended to be longer in patients with proximal and cranial cystic duct branches and had a lower success rate in patients with caudal cystic duct branches.

## 4. Discussion

In this study, we showed the most common cystic duct directions and locations were the proximal, right, and cranial branches. The accuracy of the cystic duct direction and location (proximal or distal, right or left, and cranial or caudal) was 79%, 60%, and 58% by CT, and 68%, 55%, and 58% by MRCP, respectively. Additionally, we revelated the new finding that patients showing gallbladder contrast on cholangiography underwent ETGBD with a significantly shorter procedure time than those without gallbladder contrast on cholangiography.

The standard treatment for acute cholecystitis is early laparoscopic cholecystectomy. However, the presence of severe comorbidities and low performance status increase the mortality of cholecystectomy [14]. In such situations, gallbladder drainage, including ETGBD and PTGBD, is a treatment option for acute cholecystitis. ETGBD is technically challenging, and previous reports showed that the technical success rate of ETGBD was 64–100% [8], which is lower than that of PTGBD. The primary reasons for unsuccessful ETGBD include the inability to identify or cannulate the cystic duct, followed by the inability to advance the guidewire through the cystic duct. Therefore, forecasting the direction and location of the cystic duct before performing ETGBD may facilitate insertion of the guidewire into the gallbladder.

According to previous reports, the most common cystic duct directions and locations are the proximal, right, and cranial branches [15]. Maruta et al. reported that the cystic duct showed proximal, right, and cranial branches in 76%, 86%, and 80% of the cases, respectively, which is similar to our results [16]. However, no previous reports have described the diagnostic imaging ability of cystic duct anatomy in patients with acute cholecystitis before performing ETGBD.

The current study showed the accuracy of pre-procedural imaging of the cystic duct direction and location. We evaluated 145 patients with CT and 31 patients with MRCP. The accuracy of CT was 79% for proximal/distal, 60% for right/left, and 58% for cranial/caudal. The accuracy of MRCP was 68% for proximal/distal, 55% for right/left, and 58% for cranial/caudal. To the best of our knowledge, this is the first report to evaluate the accuracy of pre-procedural CT and MRCP in patients who underwent ETGBD for acute cholecystitis. A previous report describing MRCP findings obtained pre-cholecystectomy showed that the sensitivity of MRCP for an abnormal cystic duct was just 50% [17]. Our data revealed that pre-procedural MRCP and pre-procedural CT in patients with acute cholecystitis did not have sufficient accuracy. Though the reason of low accuracy of cystic duct direction and location by CT and MRCP has not been clarified, inflammation around the gallbladder might affect the poor detection of the cystic duct.

The effects of the cystic duct direction and location on successful ETGBD are under discussion. Cao et al. reported that the technical success of cannulation of the gallbladder had no significant influence on the direction and location of the cystic duct [18]. On the other hand, Yane et al. noted that patients with a right/cranial cystic duct had a high technical success rate against other directions of the cystic duct [19]. In addition, Maruta et al. showed that the cystic duct direction (caudal) and location (proximal) were the factors affecting the technical failure of ETGBD [16]. Our results could not reveal the effect of cystic duct direction and location on the technical success of ETGBD. However, although there was no statistically significant difference, the ETGBD success rate tended to be lower in patients with caudal cystic duct branches. Additionally, the cystic duct direction and location did not affect the procedure time and cystic duct injury during ETGBD in this study. However, although there was no statistically significant difference, the procedure time tended to be longer in patients with proximal and cranial cystic duct branches.

We evaluated the relationship between the presence of gallbladder contrast by cholangiography and the outcomes of ETGBD. Patients who showed gallbladder contrast on cholangiography required a significantly shorter procedure time for ETGBD and had a lower rate of cystic duct injury. These results suggest that the procedure can be performed more easily and safely in patients showing gallbladder contrast on cholangiography.

The current study had several limitations. First, it was a nonrandomized, retrospective study conducted at a single center. Second, all ETGBDs were performed by a specialist in ERCP and ETGBD. Therefore, the results were not generalizable. Finally, even though the CT and MRCP images were checked by three experts of pancreatobiliary endoscopy, observer biases could not be ruled out.

## 5. Conclusions

Pre-procedural assumptions of cystic duct direction and location by CT or MRCP before ETGBD were difficult in patients with acute cholecystitis. The cystic duct direction and location might not affect the technical success, procedure time, or cystic duct injury rate of ETGBD. Patients showing gallbladder contrast on cholangiography required a significantly shorter time for ETGBD and had a lower rate of cystic duct injury.

## Figures and Tables

**Table 1:** Baseline characteristics.

Characteristic	*n* = 145
Male:Female	76:69
Median age, years (range)	77 (12–96)
Couse of cholecystitis	
Gallstone	119 (82%)
Metal stent	6 (4%)
Malignant obstruction	8 (6%)
Other	12 (8%)
Severity of cholecystitis	
Mild	69 (48%)
Moderate	55 (38%)
Severe	21 (14%)
Pre-ERCP images	
Contrast-enhanced CT	145 (100%)
MRCP	29 (20%)
Stone impaction	
In cystic duct	17 (12%)
In gall neck	21 (14%)
CBD stones	46 (32%)

ERCP, endoscopic retrograde cholangiopancreatography; CT, computed tomography; MRCP, magnetic resonance cholangiopancreatography; CBD, common bile duct.

**Table 2:** Cholangiography and ERCP procedures.

	*n* = 145
Contrast on cholangiography	
without contrast	85 (59%)
Only cystic duct contrast	31 (21%)
Cystic duct and gallbladder contrast	29 (20%)
Cystic duct direction and location	
Proximal/distal	114/31
Right/left	101/44
Cranial/caudal	141/4
Procedure time, min (range)	50 (13–129)
Technical success	127/145 (88%)
Cystic duct injury	16 (11%)
Injury by guidewire	11 (8%)
Injury by cannula	5 (3%)

ERCP, endoscopic retrograde cholangiopancreatography.

**Table 3:** Accuracy of cystic duct direction and location by CT and MRCP.

	CT *n* = 145	MRCP *n* = 31
Proximal or distal, *n* (%)	114/145 (79)	21/31 (68)
Right or left, *n* (%)	87/145 (60)	17/31 (55)
Cranial or caudal, *n* (%)	84/145 (58)	18/31 (58)

CT, computed tomography; MRCP, magnetic resonance cholangiopancreatography.

**Table 4:** Relationship between pre-procedural images and ETGBD.

		Presence of the Factor		
	Factor	Yes	No	*p*-Value
Procedure time, min (range)	with gallbladder contrast	43 (13–93)	53 (19–129)	0.023
	proximal cystic duct branch	53 (13–129)	43 (21–96)	0.050
	right cystic duct branch	50 (13–129)	50 (20–100)	0.40
	cranial cystic duct branch	77 (47–125)	50 (13–129)	0.073
Technical success of ETGBD, *n* (%)	with gallbladder contrast	27/29 (93)	100/116 (86)	0.53
	proximal cystic duct branch	101/114 (89)	26/31 (84)	0.54
	right cystic duct branch	87/101 (86)	40/44 (91)	0.59
	cranial cystic duct branch	125/141 (89)	2/4 (50)	0.075
Cystic duct injury, *n* (%)	with gallbladder contrast	0/29 (0)	16/116 (14)	0.042
	proximal cystic duct branch	10/114 (9)	6/31 (19)	0.11
	right cystic duct branch	11/101 (11)	5/44 (11)	1.0
	cranial cystic duct branch	15/141 (11)	1/4 (25)	0.38

ETGBD, endoscopic transpapillary gallbladder drainage.

## Data Availability

The data are not publicly available for the viewpoint of protecting personal information.

## Abbreviations

CT	computed tomography
PTGBD	percutaneous transhepatic gallbladder drainage
ETGBD	endoscopic transpapillary gallbladder drainage
MRCP	magnetic resonance cholangiopancreatography
ERCP	endoscopic retrograde cholangiopancreatography
Fr	French
EGBS	endoscopic gallbladder stenting
EBS	endoscopic biliary stenting
ENBD	endoscopic nasobiliary drainage
